# Detecting riboSNitches with RNA folding algorithms: a genome-wide benchmark

**DOI:** 10.1093/nar/gkv010

**Published:** 2015-01-23

**Authors:** Meredith Corley, Amanda Solem, Kun Qu, Howard Y. Chang, Alain Laederach

**Affiliations:** 1 Department of Biology, University of North Carolina at Chapel Hill, Chapel Hill, NC 37599, USA; 2 Curriculum in Bioinformatics and Computational Biology, University of North Carolina at Chapel Hill, Chapel Hill, NC 27599, USA; 3 Program in Epithelial Biology, Stanford University School of Medicine, Stanford, CA 94305, USA; 4 Howard Hughes Medical Institute, Stanford University, Stanford, CA 94305, USA

## Abstract

Ribonucleic acid (RNA) secondary structure prediction continues to be a significant challenge, in particular when attempting to model sequences with less rigidly defined structures, such as messenger and non-coding RNAs. Crucial to interpreting RNA structures as they pertain to individual phenotypes is the ability to detect RNAs with large structural disparities caused by a single nucleotide variant (SNV) or riboSNitches. A recently published human genome-wide parallel analysis of RNA structure (PARS) study identified a large number of riboSNitches as well as non-riboSNitches, providing an unprecedented set of RNA sequences against which to benchmark structure prediction algorithms. Here we evaluate 11 different RNA folding algorithms’ riboSNitch prediction performance on these data. We find that recent algorithms designed specifically to predict the effects of SNVs on RNA structure, in particular remuRNA, RNAsnp and SNPfold, perform best on the most rigorously validated subsets of the benchmark data. In addition, our benchmark indicates that general structure prediction algorithms (e.g. RNAfold and RNAstructure) have overall better performance if base pairing probabilities are considered rather than minimum free energy calculations. Although overall aggregate algorithmic performance on the full set of riboSNitches is relatively low, significant improvement is possible if the highest confidence predictions are evaluated independently.

## INTRODUCTION

Accurate RNA structure prediction remains a contemporary challenge in the field of bioinformatics (1–3). The most common approach for predicting RNA structure is minimizing a free energy function derived from thermodynamic parameters for base pairing and stacking energies (4–6). Extensive benchmarking of such algorithms has contributed to significant advances in our ability to correctly predict the secondary structure of RNA (7–9). Most improvements in RNA structure prediction have focused on highly structured transcripts, i.e. RNAs that have evolved to adopt a narrow range of well-defined conformations often conferring a specific activity such as self-splicing (10–13).

Many messenger RNAs (mRNAs) and non-coding RNAs (ncRNAs) are not evolved to adopt rigidly defined structures, in general adopting an ensemble of diverse conformations. Minimum free energy (MFE) structure prediction strategies are therefore not well suited for these types of RNAs (14,15). Accurate prediction of the accessibility of specific sequence motifs in transcripts plays a decisive role in understanding post-transcriptional regulation, as transcript secondary structure can impact the binding of RNA binding proteins, ribosomes and miRNAs (16–21). However, given that these RNAs adopt a wide range of structures, traditional structural benchmarking is complicated by the fact that experimental techniques to determine an ensemble of structures do not exist for large RNAs. An alternative strategy is to benchmark folding algorithms’ performance in predicting the perturbation on the structural ensemble by particular mutations (22). A comprehensive and consistent RNA structure data set on a large number of mutations in mRNA transcripts was not available until very recently (23).

The advent of transcriptome wide RNA structure probing, and in particular the development of PARS (parallel analysis of RNA structure), provides us with the most comprehensive mRNA and ncRNA benchmark data set available to date (24,25). PARS gathers RNA sequencing reads from transcripts processed by one of the two nucleases with diametric affinities for structured versus unstructured regions of RNA. The information from the two nucleases is combined to produce scores reflecting the degree of base pairing at single nucleotide resolution (24). While other important studies have probed RNA structure at a large scale (26–29), the recent PARS data set is the first to have detected riboSNitches genome wide (23). The comparative structural analysis of a human family trio's (mother, father, child) transcriptome structure by PARS has identified almost 2000 riboSNitches (23) in the human transcriptome. A riboSNitch is an element of RNA that changes structure if a specific single nucleotide variant (SNV) is present (14–15,22–23,30). Although the majority of riboSNitches have no known phenotypic consequence, specific examples of changes in transcript structure near regulatory regions in mRNAs are associated with human disease (14–15,30).

Accurately predicting the extent to which an SNV or mutation disrupts RNA structure is important for the interpretation of personal genomes, since the structural consequences of sequence variants on an individual's transcripts can impact overall phenotypic characteristics (31,32). Even though the vast majority of riboSNitches will likely have limited phenotypic consequences, a structural prediction interpreted in the context of known functional motifs in a transcript can predict function (14,23,30). A series of algorithms have recently been proposed to tackle this challenge (15,33–35). Traditional MFE class algorithms can also be used to predict riboSNitches, although previous benchmarks on *in vitro* transcribed structured RNAs suggest they overestimate the potential structural disruption of an SNV (22,36–37). The most recent algorithms for predicting the structural disruption of an SNV have therefore focused on analyzing changes in base pairing probability matrix (BPPM) computed from partition function analysis of the Boltzmann suboptimal ensemble (38–40). The benchmark carried out below uses the PARS data set to identify the best algorithmic practices for riboSNitch detection. Furthermore, the performance trends of all prediction algorithms on subsets of differentially validated riboSNitches reveal the relative importance of thermodynamically controlled base pairing in mRNA structure change. Our analysis illustrates the significant remaining computational challenge of riboSNitch prediction and the importance of biological context when making these predictions.

## MATERIALS AND METHODS

### The benchmark data set

The PARS data set tested a total of 12233 specific SNV-transcript pairs, with 1907 of these determined to be riboSNitches. For consistency in benchmarking algorithms we considered the 50 bases 5′ and 3′ around each SNV as standard input sequence for folding prediction, or 101 bases total. SNV-transcript pairs that contain less than 50 nucleotides between the SNV and the transcription start site were excluded. In many cases, one SNV tested with several different transcripts has the same surrounding sequence in each isoform. These SNVs were condensed into one entry to ensure a set of non-redundant sequences. This curated set of SNV-transcript pairs contains 1058 riboSNitches and 5469 non-riboSNitches. RiboSNitches were organized into ‘symmetric’ and ‘asymmetric’ categories based on whether or not pairwise comparisons between mother, father and child consistently indicated a riboSNitch in the presence of different genotypes. RiboSNitches that were further validated with allele-specific mapping were also added to the ‘validated’ category, and riboSNitches that were validated with chemical probing were added to the ‘probed’ category. For sequences corresponding to multiple SNV-transcript pairs, the presence of one pair qualifying as a riboSNitch was enough to consider the sequence of a riboSNitch in this benchmark. Likewise, an SNV-transcript pair categorized as a ‘symmetric’, or ‘validated’ or ‘probed’ riboSNitch was sufficient to place the sequence into those categories. Sequences for riboSNitches and the matched 1058 non-riboSNitches are provided as text files. RiboSNitch sequences are organized according to category.

### Benchmark design and distance metrics

Structure prediction programs were tested on the sequences containing each allele for every riboSNitch and non-riboSNitch. Non-riboSNitch sets were matched in size to each riboSNitch set to reduce computational costs. As a strategy for matching non-riboSNitches and riboSNitches in terms of their experimental validation, a non-riboSNitch set was matched to a riboSNitch set of size *n* by selecting the top *n* non-riboSNitches according to their false discovery rate (FDR)-adjusted *P*-values from PARS comparisons. Since each RNA has *P*-values on three potential comparisons—mother versus father, mother versus child and father versus child—the *P*-value used here is the average of the comparisons.

The Unix commands used for each algorithm are listed in Supplementary Table S4. The ‘specialized’ algorithms directly score the distance between sequence pairs. SNPfold (14) scores with a Pearson correlation coefficient, RNAsnp (34) returns a *P*-value on Euclidean distance, remuRNA (35) measures the relative entropy between two RNAs and RNAmute (41) measures the edit distance between MFE structures. For algorithms that do not intrinsically compare the structures of sequences between two RNA variants, predictions on dot bracket structures or BPPMs were compared for each allele. All the general algorithms except CONTRAfold (42) and CentroidFold (43) return an MFE structure as their dot bracket structure, so for simplicity dot bracket structures are referred to as MFE structures in this benchmark. CentroidFold, CONTRAfold, RNAfold (6), RNAstruture (44) and UNAFold (45) are capable of returning both MFE structures and BPPMs. MC-Fold (46) and RNAmutants (47) return only MFE structures. MFE structures were compared with the RNAdistance function from ViennaRNA 2.1.1 and BPPMs were compared with RNApdist. The RNApdist function used here is a modified version of the RNApdist function implemented by ViennaRNA (6,48). Essentially, base pairing probability differences are summed without performing an alignment of the BPPMs. The distance between base pair probability matrices of sequences 1 and 2 is given by

\begin{equation*} \mathop \sum \limits_{i = 1}^n 1 - \left( {\sqrt {p1_i^( \,p2_i^( } + \sqrt {p1_i^) \,p2_i^) } + \sqrt {p1_i^\circ \,p2_i^\circ}} \right) \end{equation*}

where *n* is the number of bases and

\begin{equation*} p_i^( = \sum\limits_{n \ge j {>} i} {p_{ij}} \end{equation*}

\begin{equation*} p_i^) = \sum\limits_{1 \le j {<} i} {p_{ij} } \end{equation*}

\begin{equation*} p_i^\circ = 1 - p_i^( - p_i^) \end{equation*}

$p_{ij}$ is the probability of base *i* being paired with base *j*. $p_i^( ,p_i^)$ and $p_i^\circ$ are the probabilities of base *i* being upstream paired, downstream paired and unpaired, respectively, for BPPMs 1 and 2. Note that this modification on RNApdist assumes that the sequences being compared have the same length. The ViennaRNA implementation of the RNApdist function was used for benchmarking RNAfold (which is the main folding algorithm in the ViennaRNA package).

### Receiver operator curve analysis

The distance scores predicted for a riboSNitch set and matched non-riboSNitch set were compared with receiver operating characteristic analysis. Receiver operator curves (ROCs) were constructed and their areas measured using the R package pROC version 1.7.3 (49). ROC curves were constructed in this way for every RNA folding algorithm across all riboSNitch categories.

Twenty five and five percent subsets of all riboSNitches were selected based on riboSNitches with the lowest average FDR-adjusted *P*-values. Non-riboSNitch sets were matched to these subsets using the method applied to the other riboSNitch categories described above. ROC analysis was performed on these subsets as before. Results are shown in Supplementary Table S1.

To test the n% tails of a riboSNitch category—25% and 5% tails tested here—all of a program's scores from riboSNitches and non-riboSNitches were combined to determine the threshold values that mark the middle 100-n% of the distribution. Scores above and below these threshold values were then selected from riboSNitch and non-riboSNitch score sets separately. ROC analysis was then performed on these selected values as described previously. Score distributions and 5% tails for every algorithm are illustrated in Supplementary Figures S3A and B. Distributions were graphed with the density function in R 3.1.1.

The 95% confidence interval was calculated for each area under the curve (AUC) value using the DeLong method in the pROC package (49,50). Essentially, 95% confidence intervals are calculated as AUC±1.96*s*, where *s* is the standard deviation of the given AUC. Any comparisons between AUC values generating a *P*-value were completed with the pROC package roc.test function conducting a one-tailed test with DeLong's test for two ROC curves (49,50).

An ROC curve's ‘best’ point was considered to be the point closest to the top left corner of the graph. The threshold yielding the best point as well as the specificity and sensitivity values of the point were listed for every ROC curve (Supplementary Table S2). These were determined with the pROC R package coords function (49). Thresholds in the ‘probed’ category could be reasonably used as cutoff scores for riboSNitch detection.

## RESULTS

### Experimental benchmark criteria

**Figure 1.**
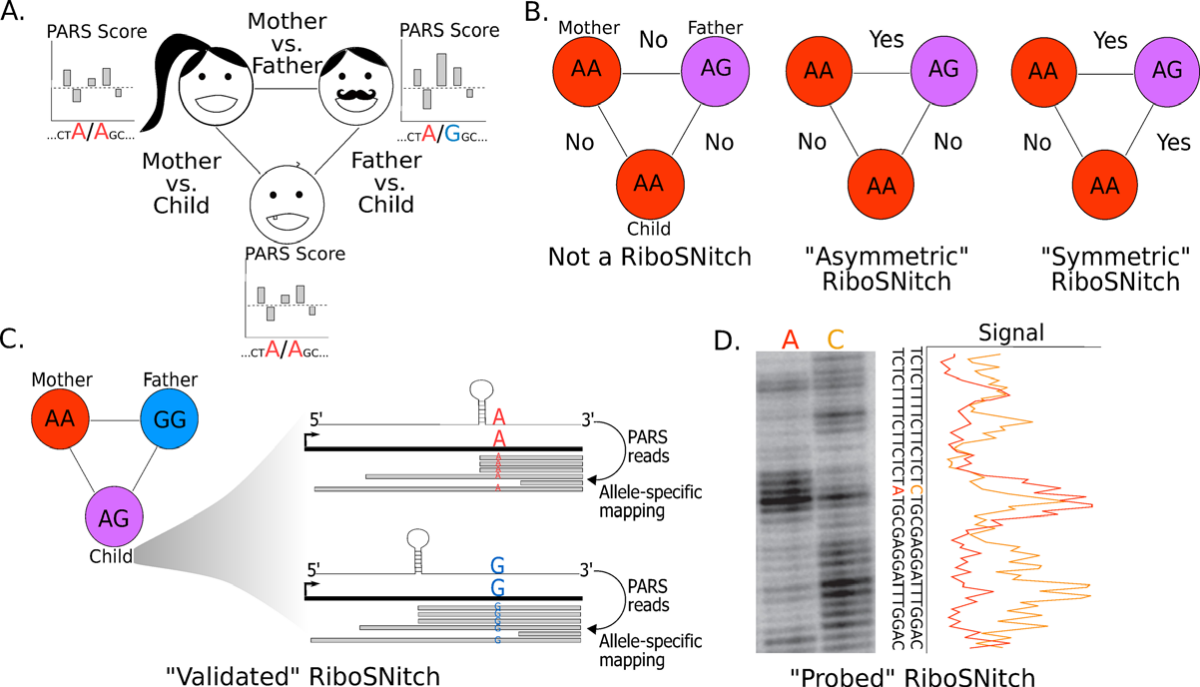
(**A**) Genome-wide experimental riboSNitch discovery using PARS data on a family trio (father, mother, child) as carried out in (23). PARS scores near loci with different genotypes between individuals are quantitatively compared to identify significant differences in structure around the SNV. (**B**) Diagrams reflect the format in (A), where genotypes refer to the genotypes of the mother, father and child at a single locus. ‘Yes’ and ‘No’ indicate whether or not local PARS score comparisons between the individuals indicated a structural difference—a riboSNitch. Two classes of riboSNitches are defined, ‘symmetric’ and ‘asymmetric’. In the ‘symmetric’ class, all genotypic differences correspond to structural differences in the PARS data, indicating consistent RNA structure changes. The ‘symmetric’ riboSNitch therefore has a higher degree of experimental confidence than its ‘asymmetric’ counterpart. (**C**) An additional level of validation is possible for riboSNitches for which the parents are homozygous different by carrying out allele-specific mapping in the heterozygous child. This validation involves assigning PARS reads (gray bars) from the child in an allele-specific manner to confirm the transcript structural profile difference between the alleles. (**D**) SHAPE (Selective 2′ Hydroxyl Acylation by Primer Extension) chemical probing validation by *in vitro* SHAPE of a 150-nucleotide fragment of the 5′ UTR of MRPS21 (Mitochondrial Ribosomal Protein S21) indicates different reactivity profiles for the A and C alleles of rs1050818. Fifteen nucleotides 5′ and 3′ of the SNV are shown in the gel and corresponding quantification of the data. Such independent chemical probing was previously performed on 11 RNAs and provides the highest confidence riboSNitches for our benchmark data set.

The experimental data for the benchmark are based on a PARS analysis of three related individuals (mother, father and child) from the 1000 Genomes Project (23). PARS measures the differential reactivity of each nucleotide in a folded RNA to the V1 and S1 RNases which selectively cleave double- and single-stranded regions, respectively (51). Thus the PARS score for each nucleotide is correlated with the extent of base pairing (24). By comparing the PARS scores at loci in individuals with different alleles (Figure 1A) it is possible to detect riboSNitches. Given that these experiments are carried out in a genome-wide manner, thousands of putative riboSNitches were identified (37). The precise number of riboSNitches identified in such a genome-wide screen depends on the threshold in PARS scores used to call a structural difference. A careful analysis of PARS score differences identified 1907 loci in the human genome as riboSNitches and 10326 loci where no significant change was observed (non-riboSNitches) (23). These data form the basis for our benchmark study.

A riboSNitch in the context of the PARS data set was identified by pairwise comparison of PARS score profiles for transcripts from two individuals in the family trio. Three individual-to-individual comparisons are therefore possible as illustrated in Figure 1B. We used this redundancy to identify subsets of riboSNitches with differing levels of experimental confidence. In the most consistent case, the ‘symmetric’ riboSNitch, PARS score profiles are significantly different in all cases comparing individuals with different genotypes. In certain cases, however, not every genotypic difference results in a significant PARS profile difference; we consider these cases ‘asymmetric’ riboSNitches.

An alternative approach for validating a riboSNitch using the PARS data involves allele-specific mapping at heterozygous loci in the child data set. This type of validation was performed at loci where the parents are homozygous different with a necessarily heterozygous child, as diagramed in Figure 1C. Successful validation also requires that the significant structure change be 3′ of the SNV. This is a consequence of PARS library preparation, in which only fragments 5′ of the endonuclease cut site are sequenced; only sequencing reads that include the SNV can be mapped in an allele-specific manner. Allele-specific analysis of the PARS data validated 115 riboSNitches (23) and we refer to these riboSNtiches as ‘validated’. The most rigorous form of validation is independent chemical and enzymatic structure probing on *in vitro* transcribed constructs for each allele, as illustrated on Figure 1D (52–54). This involves a separate experiment, and 11 riboSNitches were further validated in this way (23). This set of riboSNitches is referred to as ‘probed’ for the purposes of this benchmark. The categorization proposed here yields subsets of riboSNitches for use in our benchmark with differing levels of experimental validation. We refer to the entire set of riboSNitches as the ‘all’ data set or category. Additional filtering was applied to the ‘all’ set of 1907 reported (23) riboSNitches to ensure a completely non-redundant data set. A riboSNitch in the PARS data set is identified as an SNV in a specific transcript isoform. In this study, we use sequences centered on these SNVs to test folding algorithms. However, many transcripts have isoforms with the same window (±50 nt) of sequence around a particular SNV. As a result, we benchmarked on the 1058 unique sequence subset of the 1907 reported riboSNitches. The numbers of unique riboSNitches and corresponding non-riboSNitches for each level of experimental validation are reported in Table 1. Non-riboSNitch set sizes were matched to each riboSNitch category size in order to reduce computational costs as well as to match non-riboSNitches and riboSNitches in terms of their experimental validation (methods).

**Table 1.** The size of riboSNitch sets and non-riboSNitch sets in each category

	Probed	Validated	Symm	Asymm	All	25% tails^a^	5% tails^a^
riboSNitch	11	63	223	835	1058	260–288	47–59
Non-riboSNitch	11	63	223	835	1058	240–268	46–79

^a^Set sizes from the MFE algorithms were not included in this range.

### Algorithmic performance analysis

**Figure 2.**
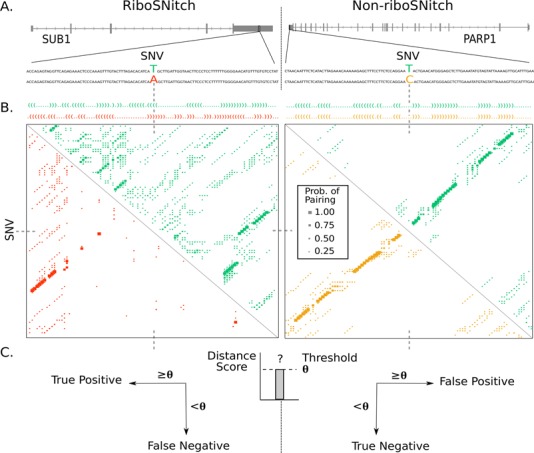
RiboSNitch prediction and benchmarking strategy. (**A**) To benchmark, all the prediction algorithms estimate structure features for both alleles of an RNA. This is done for both the riboSNitch sets and non-riboSNitch sets. The riboSNitch in this example is the sequence flanking an SNV in the 3′ UTR of SUB1 (activated RNA polymerase II transcriptional co-activator p15 or SUB1 homolog) that yields a differential PARS score between alleles. The non-riboSNitch is the sequence flanking an SNV in the 3′ UTR of PARP1 (poly [ADP-ribose] polymerase 1) where no significant PARS score differences were measured. Alleles are color-coded with green and red for the T and A riboSNitch alleles and green and yellow for the T and C non-riboSNitch alleles. (**B**) RNAfold (6) predicted minimum free energy structures and base pairing probability matrices for the riboSNitch and non-riboSNitch alleles. In this example, the prediction is correct in that the riboSNitch T (green) and A (red) alleles show a large difference in predicted base pairing probabilities while the non-riboSNitch T (green) and C (yellow) alleles do not. (**C**) The difference between alleles’ structures is measured to produce a distance score. Whether the riboSNitch is considered a True Positive (TP) or False Negative (FN) and the non-riboSNitch a True Negative (TN) or False Positive (FP) depends upon the score threshold used. The distance scores between alleles are used as thresholds to perform receiver operator curve (ROC) analysis (57) and evaluate predictive performance.

Given the unprecedented number of riboSNitches discovered in the human genome, our benchmark has the potential to broadly evaluate the performance of prediction algorithms. A general strategy for riboSNitch prediction and our approach to benchmarking is summarized in Figure 2. A structure prediction is made on RNA sequences containing both alleles (Figure 2A) for subsequent comparison. To benchmark the algorithms, predictions are made for sequences identified as riboSNitches and those where no experimental structure change is observed. For example, in Figure 2A, a T/A SNV in the 3′ untranslated region (UTR) of SUB1 (activated RNA polymerase II transcriptional co-activator p15 or SUB1 homolog) is a riboSNitch since PARS scores differ at this locus, indicating a structure difference between the two alleles. The T/C SNV in the 3′ UTR of PARP1 (poly [ADP-ribose] polymerase 1) does not alter structure in the PARS data and is therefore a non-riboSNitch. The structures of each variant are compared (Figure 2B), and, in this example, RNAfold (6) correctly predicts that the base pairing probabilities computed for each allele are very different for the riboSNitch, and nearly identical for the non-riboSNitch (Figure 2B, left versus right panel).

In choosing RNA structure prediction algorithms to test in the benchmark we opted for a variety of algorithms. We differentiate prediction algorithms into two broad classes: specialized (i.e. algorithms specifically engineered to predict the effects of mutations on RNA structure) and general (i.e. algorithms that are developed to predict RNA structure). Specialized algorithms generally report a score (or confidence) for a predicted difference between mutant RNAs. For non-specialized algorithms a scoring metric to compare base paring probabilities or MFE predictions is required. We used RNAdistance and a custom implementation of RNApdist, algorithms implemented in the ViennaRNA package, to carry out these comparisons and quantify structural distances (6,55–56). This allows us to define a variable threshold over predictions on riboSNitches and non-riboSNitches for each algorithm (Figure 2C) and perform ROC analysis to benchmark performance (57). By performing ROC analysis for each algorithm on the subsets of riboSNitches with differing levels of experimental evidence (Figure 1), we are also able to indirectly evaluate the relative importance of the different types of experimental validation.

**Figure 3.**
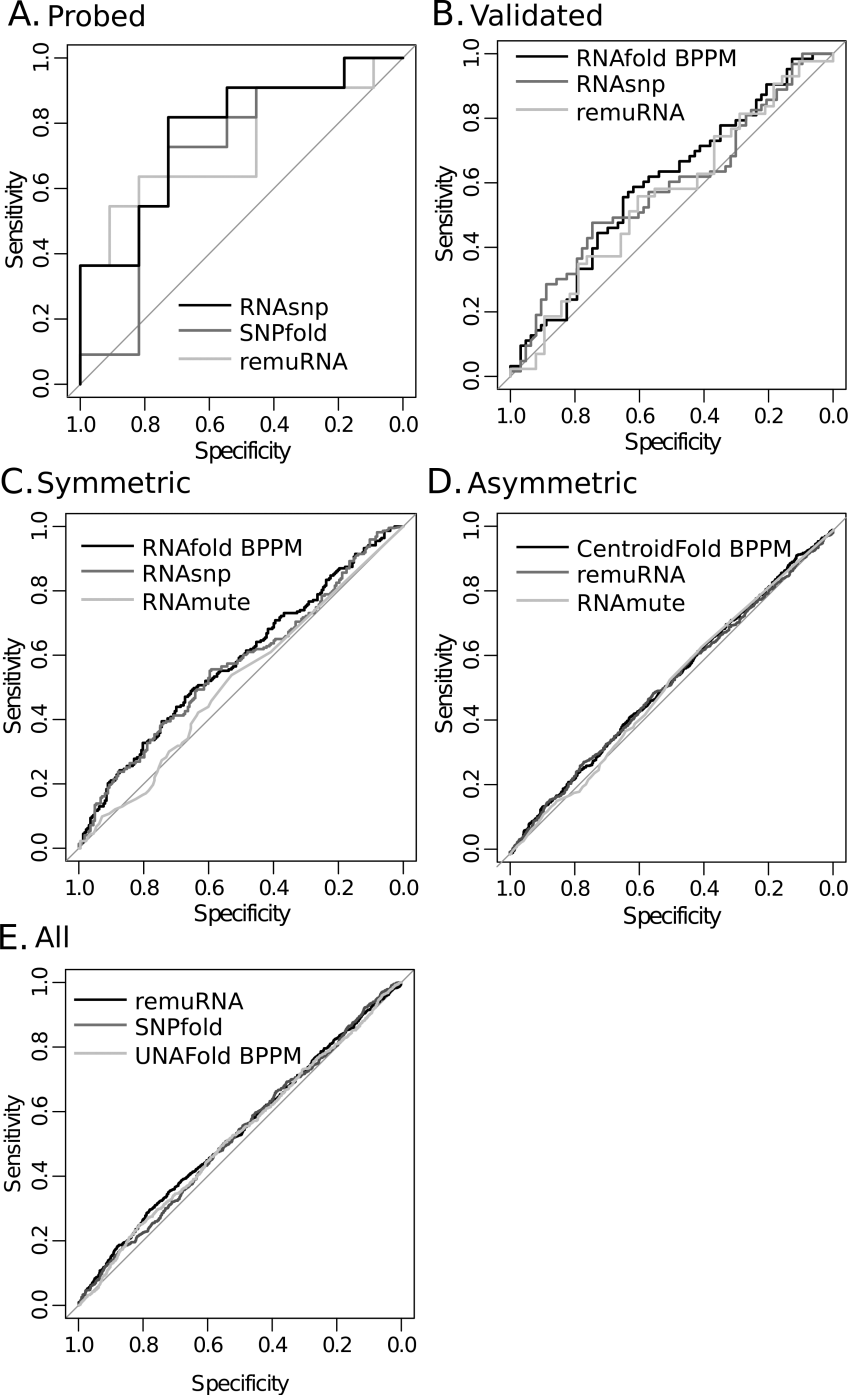
Representative ROC curves for best (black), mid (dark gray) and low (light gray) performing algorithms on different categories of riboSNitches in the benchmark data. (**A**) Representative performance riboSNitches validated with *in vitro* chemical probing. (**B**) Representative performance on allele-specific PARS mapped riboSNitches or ‘validated’ riboSNitches. (**C**) Representative performance on ‘symmetric’ riboSNitches. (**D**) Representative performance on ‘asymmetric’ riboSNitches. (**E**) Representative performance on all riboSNitches.

Representative ROC curves for the five different levels of experimental riboSNitch validation are shown in Figure 3A–E, respectively. Large differences in performance are observed between algorithms (especially with the ‘probed’ riboSNitches; Figure 3A); the specialized algorithms in most cases outperform the general class of algorithms (Tables 2 and 3). This is unsurprising given that the general algorithms were not specifically designed for riboSNitch prediction; however, some of the general algorithms still show comparable performance to the specialized (Table 3, bold values). The best prediction performance is observed on the most highly validated subset of riboSNitches, i.e. those validated independently by *in vitro* chemical and enzymatic probing. The prediction performance of the specialized algorithms on the *in vitro* validated (or ‘probed’) data is on par with, and in some cases higher than, what was observed in a previous benchmark on structured RNAs performed in 2012 (22). Recent algorithmic developments, and in particular the analysis of local structure change performed in RNAsnp, appear to improve riboSNitch predictions for mRNA and ncRNAs (35).

**Table 2.** ROC results^a^ for specialized algorithms designed to predict riboSNitches

Software	Prediction program^b^	Probed	Validated	Symm	Asymm	All	25% tails	5% tails
remuRNA (35) 03Nov2012	McCaskill-remuRNA	**0.736** (0.514–0.957)	0.557 (0.456–0.658)	0.543 (0.49–0.597)	0.524 (0.497–0.552)	0.537 (0.513–0.562)	0.574 (0.525–0.622)	0.567 (0.455–0.679)
RNAmute (41) 1.0	RNAfold 1.4	0.512 (0.258–0.767)	0.568 (0.469–0.667)	0.516 (0.464–0.568)	0.517 (0.49–0.544)	0.511 (0.487–0.535)	0.510 (0.484–0.536)	0.503 (0.487–0.519)
RNAsnp (34) 1.1	RNAfold 1.1	**0.777** (0.573–0.98)	0.583 (0.483–0.684)	0.568 (0.515–0.621)	0.529 (0.501–0.557)	0.533 (0.508–0.558)	0.583 (0.535–0.631)	0.658 (0.556–0.761)
SNPfold (14) 1.01	RNAfold 2.1.1	**0.703** (0.466–0.939)	0.581 (0.48–0.681)	0.571 (0.518–0.624)	0.520 (0.493–0.548)	0.528 (0.504–0.553)	0.591 (0.543–0.639)	**0.736** (0.638–0.835)

^a^Results are reported as the area under the curve (AUC) for each ROC curve with 95% confidence intervals underneath. Top performers are in bold.

^b^The underlying program that this software bases its structure prediction on.

**Table 3.** ROC results^a^ for general RNA folding algorithms

Software	Structure type^b^	Probed	Validated	Symm	Asymm	All	25% tails	5% tails
CentroidFold (43) 00.0.9	BPPM	0.579 (0.324–0.833)	0.561 (0.46–0.662)	0.569 (0.516–0.622)	0.529 (0.502–0.557)	0.532 (0.507–0.556)	0.596 (0.548–0.645)	0.637 (0.528–0.746)
	MFE	0.512 (0.257–0.768)	0.574 (0.474–0.674)	0.575 (0.523–0.627)	0.524 (0.497–0.552)	0.534 (0.51–0.558)	0.540 (0.509–0.57)	0.510 (0.489–0.531)
CONTRAfold (42) 2.02	BPPM	0.463 (0.195–0.73)	0.562 (0.461–0.664)	0.567 (0.514–0.62)	0.528 (0.500–0.556)	0.535 (0.510–0.559)	0.562 (0.513–0.612)	0.613 (0.504–0.72)
	MFE	0.347 (0.098–0.596)	0.557 (0.456–0.658)	0.548 (0.495–0.601)	0.513 (0.485–0.54)	0.515 (0.490–0.539)	0.514 (0.479–0.549)	0.511 (0.485–0.537)
MC-Fold (46) 17Mar2008	BPPM	NA	NA	NA	NA	NA	NA	NA
	MFE	0.424 (0.135–0.712)	0.460 (0.331–0.589)	0.478 (0.417–0.539)	0.497 (0.465–0.53)	0.493 (0.464–0.522)	0.493 (0.459–0.531)	0.493 (0.474–0.53)
RNAfold (6) 2.1.1	BPPM	**0.686** (0.441–0.931)	0.597 (0.498–0.697)	0.581 (0.528–0.634)	0.525 (0.497–0.553)	0.534 (0.509–0.559)	0.589 (0.540–0.637)	**0.707** (0.606–0.808)
	MFE	0.471 (0.21–0.732)	0.554 (0.455–0.654)	0.531 (0.48–0.585)	0.515 (0.487–0.541)	0.519 (0.493–0.541)	0.518 (0.491–0.545)	0.503 (0.485–0.521)
RNAmutants (47) 2.0	BPPM	NA	NA	NA	NA	NA	NA	NA
	MFE	0.517 (0.255–0.778)	0.474 (0.374–0.574)	0.504 (0.451–0.556)	0.510 (0.483–0.537)	0.509 (0.485–0.533)	0.501 (0.475–0.528)	0.493 (0.476–0.51)
RNAstructure (44) 5.6	BPPM	**0.612** (0.358–0.865)	0.578 (0.478–0.678)	0.567 (0.513–0.62)	0.527 (0.499–0.554)	0.536 (0.511–0.560)	0.553 (0.504–0.602)	**0.622** (0.514–0.731)
	MFE	0.413 (0.152–0.674)	0.545 (0.444–0.645)	0.533 (0.481–0.585)	0.525 (0.498–0.552)	0.527 (0.503–0.551)	0.535 (0.508–0.561)	0.510 (0.493–0.527)
UNAFold (45) 3.8	BPPM	0.471 (0.21–0.732)	0.537 (0.435–0.639)	0.548 (0.494–0.601)	0.524 (0.496–0.551)	0.526 (0.502–0.551)	0.528 (0.478–0.577)	0.578 (0.467–0.688)
	MFE	0.343 (0.093–0.593)	0.574 (0.474–0.673)	0.526 (0.474–0.579)	0.519 (0.492–0.546)	0.512 (0.488–0.536)	0.519 (0.492–0.547)	0.499 (0.481–0.517)

^a^Results are reported as the area under the curve (AUC) for each ROC curve with 95% confidence intervals underneath. Top performers are in bold.

^b^Structure distances were measured by comparing base pairing probability matrices (BPPMs) and/or minimum free energy (MFE) structures.

Performance benchmarks for all algorithms tested are summarized in Tables 2 and 3 along with 95% confidence intervals. While the intervals are often wide due to small sample sizes, a few consistent patterns are apparent throughout. The majority of algorithms perform best on the most experimentally validated data set. This is true of both generalized and specialized prediction algorithms. In addition, aggregate prediction accuracy of all algorithms decreases with lower levels of experimental validation. None of the algorithms have good performance on the ‘asymmetric’ and ‘all’ data sets (Figure 3E), yielding AUC values slightly greater than 0.5 (Tables 2 and 3). This is expected to some extent since the experimental FDR increases with lower levels of experimental confidence. However, it could also result from a population of RNAs in the data that are riboSNitches only *in vivo* and not purely driven by thermodynamic changes in base pairing probabilities. Since the PARS experiment was carried out on *in vivo* transcribed RNAs, it has the potential to detect such riboSNitches (23). RNA structure in the cell may differ from that in the tube for a number of reasons. Co-transcriptional folding may encourage certain structures over others (58) and small RNA (miRNA and siRNA) and protein binding to transcripts may stabilize or induce certain structural elements (59). Folding is known to depend on solvent conditions and could thus be influenced by conditions in the cell like salt concentration, pH and molecular crowding. Post-transcriptional RNA sequence modifications could allow for additional structure disruptions that are unique to the cell environment and detectable by PARS. The thermodynamic prediction algorithms used here likely fail to predict large structure changes for riboSNitches that result from these environmental contributors.

The variation of structure predictions with sequence length is an important factor to consider in interpreting these results. We found that AUC values do vary with sequence length, but are robust within certain ranges of lengths (Supplementary Figure S1). Even though AUC values can have high variance, especially in the smaller categories of riboSNitches, the general trend of AUC values decreasing from highly validated riboSNitch categories to the lowest is still evident across a range of sequence lengths, tested from 21 to 201 nucleotides (Supplementary Figure S2). Furthermore, the 95% confidence intervals on algorithms’ AUC values (Tables 2 and 3) capture most of the variation in performance observed with different sequence lengths (Supplementary Figures S1 and S2).

### Improving riboSNitch prediction performance

Both riboSNitch prediction and experimental validation require defining a threshold for what is and is not a significant change in structure. Experimentally, the threshold is based on whether the PARS signal is measurably different between genotypes (23). Our benchmark shows that higher levels of experimental validation correlate with improved prediction accuracy for most algorithms (Tables 2 and 3), indicating that the FDR in the data decreases with increasing validation. To address this possible higher FDR in the category of all riboSNitches, we tested ROC analysis on subsets of riboSNitches with the lowest *P*-values, as determined by their PARS scores. Subsets used were the best 25% and the best 5% out of all riboSNitches. The resulting ROC curves show little to no improvement in their AUC values (Supplementary Table S1). This argues against false positives as the main cause of poor predictions in the ‘all’ category. It suggests instead that a prohibitive number of the experimentally predicted riboSNitches are in fact environmental riboSNitches that do not show large structure changes driven purely by base pairing thermodynamics.

All algorithms in this benchmark predict the extent of structural change caused by an SNV. They differ in the metric used to quantify the structural change and/or the specific free-energy functions used for the prediction. The specialized algorithms report a score for each structure comparison, while for the generalized algorithms we evaluate the change in either the predicted MFE structures or BPPMs (Figure 2B). These scores are a proxy for the extent of structural disruption by the SNV. Though all the algorithms predict very similar score distributions for riboSNitches and non-riboSNitches, in many cases the non-riboSNitch score distribution is more skewed toward low distance scores or strong non-riboSNitches (Supplementary Figure S3). Consequently, the largest differences between riboSNitch and non-riboSNitch distance score distributions lie at the extremes. We therefore evaluated performance of the algorithms with just their extreme-valued predictions on the ‘all’ data set. We propose that such a strategy will mitigate some of the experimental thresholding issues that make genome-wide prediction of riboSNitches difficult. In practical terms, the algorithm is allowed to ‘opt-out’ of making a prediction if the riboSNitch predicted score is not at one of the extremes of its score distribution.

**Figure 4.**
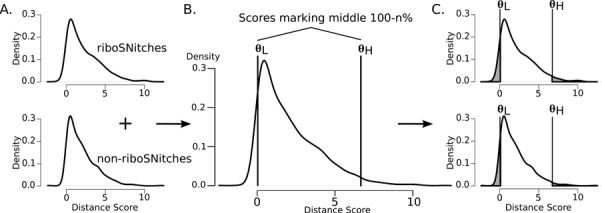
Schematic on isolating the n% tails of riboSNitch and non-riboSNitch score distributions. (**A**) riboSNitch and non-riboSNitch distance scores from a particular algorithm are temporarily combined into one larger set. (**B**) Scores that delineate the middle 100-n% of the combined distribution are determined, shown here as **θ**_L_ and **θ**_H_. (**C**) Scores that are lower than **θ**_L_ and higher than **θ**_H_ (shaded gray) are selected from the original riboSNitch and non-riboSNitch score sets for ROC analysis.

**Figure 5.**
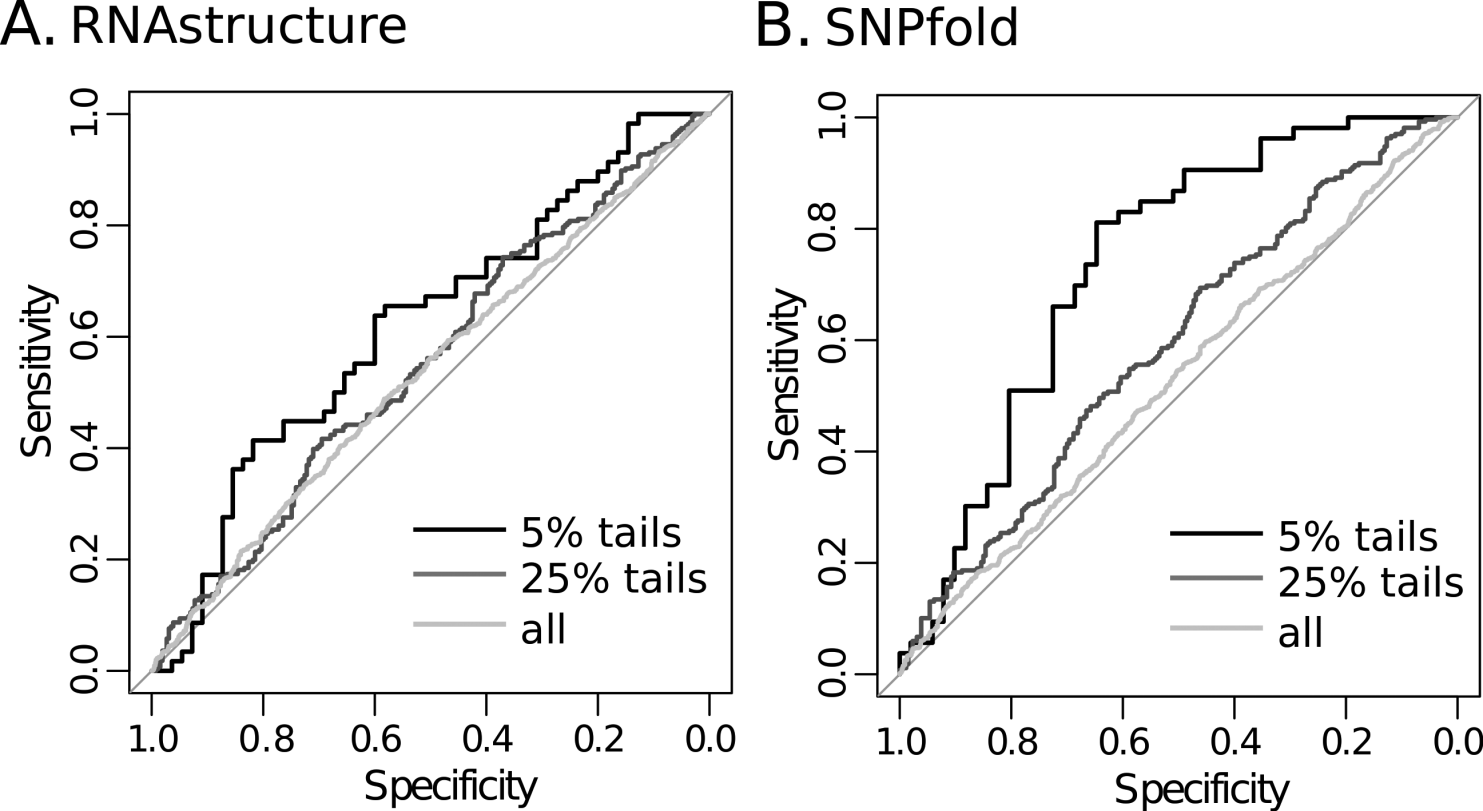
Example improvements with the n% tails strategy. (**A**) ROC curves for RNAstructure on all riboSNitches (light gray), 25% tails (medium gray) and 5% tails (black). (**B**) ROC curves for SNPfold on all riboSNitches (light gray), 25% tails (medium gray) and 5% tails (black).

To evaluate performance on these extreme-valued predictions we combined riboSNitch and non-riboSNitch distance scores from the ‘all’ category for each algorithm in order to pick the values that mark the tails occupying n% of the distribution (Figure 4A and B). Both 25% and 5% tails were tested, where the range of set sizes from scores selected in this way is listed in the last two columns of Table 1. Scores between the marker values were removed from the riboSNitch and non-riboSNitch scores to make filtered score sets for ROC analysis (Figure 4C). As can be seen in the ROC curves in Figure 5 and Tables 2 and 3, this strategy generally improves AUCs and in some cases results in prediction performance equivalent to that obtained on the ‘probed’ riboSNitch subset. RNAfold and SNPfold in particular benefit from this method, with 95% confidence intervals on their 5% tail AUC values completely outside of the confidence intervals on their ‘all’ category AUC values. Interestingly, this approach does not improve the performance of MFE-based predictions as much as BPPM-based predictions for any of the algorithms. The score distributions taken from MFE structure distances are not diverse, i.e. many of the scores are zeros, rendering this sort of filtering ineffective. However, this ‘opt-out’ method is still highly effective with BPPM-based algorithms and represents a simple strategy to improve predictions on genome-wide data sets and to identify the highest-confidence predictions.

## DISCUSSION

RNA folding is a complex process that is driven by both thermodynamic and kinetic factors (60–62). In the cell, exogenous factors including protein chaperones and small molecules interact with the RNA, further altering the folding behavior (59,63–66). In addition, RNA necessarily folds co-transcriptionally, adding another layer of kinetic complexity to the problem of accurate structure prediction (67–70). The genome-wide discovery of riboSNitches using PARS enzymatic structure probing provides an unprecedented experimental analysis of RNA structure and can significantly contribute to our understanding of the relative importance of these various factors in defining conformation (37).

Aside from CONTRAfold (42) the algorithms used in this benchmark model RNA structure relying on parameters derived from thermodynamic analysis of base pairing and base-stacking (4,71–72). Although these energy functions are still being refined, they do capture a majority of the thermodynamically important features that are known to drive RNA folding (4,72–73). The primary focus of this benchmark is to determine the best algorithms for riboSNitch prediction, though the consistent differences in performance of most algorithms on the subsets of differentially validated data reveal important lessons for RNA structure prediction in general.

Our benchmark shows that a majority of the algorithms best predict ‘probed’ category riboSNitches (Figure 3A and Tables 2 and 3). This category of validation involves independent *in vitro* transcription and folding of the RNA prior to chemical probing. Thus all sequences in the ‘probed’ data set are riboSNitches detected both *in vitro* and *in vivo.* The small size of the ‘probed’ category makes it difficult to demonstrate significant improvement compared to the other categories with a given algorithm. Nevertheless, the highest scoring algorithms have ‘probed’ category AUC values that are significantly greater than the AUC values derived from other categories. The remuRNA ‘probed’ AUC value is greater than its ‘asymmetric’ and ‘all’ AUC values (*P*-val = 0.039 and 0.047, respectively) and the RNAsnp ‘probed’ AUC is significantly greater than its ‘symmetric’, ‘asymmetric’ and ‘all’ AUC values (*P*-val = 0.032, 0.014 and 0.015, respectively). The aggregate performance of algorithms on allele-specific-validated riboSNitches (‘validated’ category) is lower than the ‘probed’ set but still consistently better than predictions on all riboSNitches (Figure 3A and B and Tables 2 and 3). ‘Symmetric’ riboSNitches are better predicted than ‘asymmetric’ (Figure 3C and D and Tables 2 and 3). These results immediately suggest that higher levels of experimental validation reduce the FDR in the PARS data, since the algorithmic prediction accuracy is likely constant. However, it may also suggest that the increasingly validated categories correspond to RNAs whose folding is governed more by thermodynamic changes in base pairing over other cellular factors. The presence of a class of thermodynamic, versus environmental, riboSNitches is more consistent with our results. Environmental riboSNitches would be poorly predicted by RNA folding algorithms, and in this benchmark, they likely blur the difference in score distributions between riboSNitches and non-riboSNitches. The ‘asymmetric’ data set, which yields the worst folding prediction performance on average, is likely enriched in RNAs whose folding is determined more by cellular environment and genetic background than by sequence-based folding dynamics, as individual variation in environment can cause RNAs to fold differently. We also cannot exclude the possibility that some of the false-positive and negative predictions are due to post-transcriptional editing (74).

RNAsnp (34) is the highest performing algorithm on the ‘probed’ data set (Table 2). This algorithm analyzes local structure changes between mutant structures, while other algorithms measure structure change over the entire length of the sequence. This result is, perhaps, unsurprising given that the riboSNitches used in this benchmark had been detected by comparing PARS scores in a small region (5 nt) around each SNV (23). While RNAsnp surpasses some of the other algorithms only marginally, considering its comparable performance, it is clear that considerations of local structure change can be successful in riboSNitch prediction.

Despite fair performance of some of the algorithms on the ‘probed’ category, performance in other categories remains poor. Two strategies were attempted to improve the performance on the ‘all’ riboSNitch category, as good performance on the most comprehensive data sets should be the ultimate goal of prediction algorithms. The first, making predictions only on riboSNitches with PARS score *P*-values in the bottom 25% and 5% of all riboSNitches, resulted in few improvements (Supplementary Table S1). This suggests that a higher experimental FDR is not solely responsible for the poor performance on the ‘all’ data set. However, we cannot exclude that the *P*-value from structural differences in the PARS data may be a poor indicator of confidence in a riboSNitch. It is important to note that the lack of improvement in AUC values with these subsets of the ‘all’ category also verifies that smaller sample sizes do not superficially improve results. Thus the better performance in the more validated categories is not simply due to their smaller sample size.

The second strategy, ROC analysis on the 5% (and 25%) tail values of riboSNitch and non-riboSNitch score distributions, resulted in marked improvements. In some cases, as in the case of SNPfold and RNAfold, performance with the 5% tails is on par with that of the chemically probed category and is significantly greater than the corresponding ‘all’ category performance (*P*-val = 5.28e-05 and 7.53e-04, respectively). The algorithms that did not improve were those that only predict MFE structures. Closer examination of MFE distance scores for ‘all’ category riboSNitches and non-riboSNitches showed sparse score distributions (i.e. a lot of zeros). Based on these results we suggest that users can make the most of genome-wide predictions by using just the top and bottom 5% of BPPM-based distance scores to indicate riboSNitches and non-riboSNitches, respectively.

It is interesting to note that changing the metric used to measure structure differences can result in better performance. All the specialized algorithms essentially apply new metrics to existing RNA folding algorithms, so by benchmarking the specialized algorithms we are testing a number of different metrics. For instance, SNPfold takes its structure predictions from RNAfold, but measures structure differences with a Pearson correlation coefficient between base pairing probabilities instead of with one of the RNAfold distance functions. Remarkably, SNPfold performs better than RNAfold (BPPM) on average with its simpler distance metric. Some of the other BPPM folding algorithms also exhibit better scores when switched to using a correlation coefficient, namely CentroidFold and CONTRAfold, while RNAstructure and UNAFold exhibit slightly lower scores (Supplementary Table S3). Different folding parameter options may affect results as well. In this study we used default parameters for many of the algorithms, but users may boost performance with specifically tailored options. For example, RNAsnp is based on the assertion that measuring local structure changes is essential for riboSNitch prediction. To this end, RNAsnp provides an option to define the minimum size of local intervals in which to measure structure change. In this benchmark we used a minimum interval length of 10, but users may choose to change this based on their sequence length or the expected scope of structural change.

Choosing BPPM predictions over MFE structure prediction results in large improvements in performance among the general algorithms. In particular, RNAfold's ‘probed’, ‘symmetric’, ‘all’, 25% tails and 5% tails categories were all significantly greater with BPPM AUC values than MFE (*P*-val = 0.044, 8.9e-03, 0.047, 6.2e-03 and 9.0e-05, respectively). RNAstructure BPPM AUC values were significantly greater than MFE values in the ‘probed’ and 5% tails categories as well (*P*-val = 0.014 and 0.024, respectively). Furthermore, MFE-based predictions on structure disruption have been shown to underperform BPPM-based predictions previously (22). Based on inspection of MFE-based distance score distributions, many of the non-riboSNitch sets yielded distance scores just as high as their matched riboSNitch sets, indicating a tendency to over-predict structure changes. However, MFE predictions on both the riboSNitch and non-riboSNitch sets often contain an abundance of zero-valued structure distance scores, representing a tendency to under-predict as well. Predictions based on MFE structures appear to be too reductive, that is, MFE predictions do not capture enough information about RNA structural ensembles to be useful for structure comparisons. Unlike other thermodynamic folding algorithms, CentroidFold does not return true MFE structures, instead choosing the structure that optimally agrees with a sequence's predicted base paring probability matrix. Interestingly, the MFE predictions from CentroidFold have the highest AUC values out of all the algorithms’ MFE results. Improved performance from representative dot bracket structures chosen in this way further underscores the importance of considering the entire Boltzmann ensemble in RNA structure prediction.

### Recommendations

Based on this benchmark we propose the following best practices for riboSNitch prediction and experimental validation.

- For single riboSNitch prediction we recommend using one of the BPPM-based specialized algorithms—remuRNA, RNAsnp or SNPfold.
- For genome-wide prediction, performance will be greatly improved if only the top and bottom 5% of SNPfold and/or RNAfold (BPPM) predictions on the particular data set are used to indicate riboSNitches and non-riboSNitches.
- Experimentally, if a structural disruption is consistently observed in multiple individuals and by allele-specific mapping, it is likely that thermodynamic changes are driving the observed structure change.

## SUPPLEMENTARY DATA

Supplementary Data are available at NAR Online.

SUPPLEMENTARY DATA
