# Supplementary material for: Detecting riboSNitches with RNA folding algorithms: a genome-wide benchmark
Source: Nucleic Acids Res. 2015 Jan 23;43(3):1859–68. doi: 10.1093/nar/gkv010 (PMC4330374; doi:10.1093/nar/gkv010)
Supplement: SUPPLEMENTARY DATA [file supp_43_3_1859__index.html]

Detecting riboSNitches with RNA folding algorithms: a genome-wide benchmark — SUPPLEMENTARY DATA 

# Detecting riboSNitches with RNA folding algorithms: a genome-wide benchmark

## SUPPLEMENTARY DATA

**Files in this Data Supplement:**

- SUPPLEMENTARY DATA
- SUPPLEMENTARY DATA
- SUPPLEMENTARY DATA
- SUPPLEMENTARY DATA
- SUPPLEMENTARY DATA
- SUPPLEMENTARY DATA
- SUPPLEMENTARY DATA
