# Supplementary material for: Detecting riboSNitches with RNA folding algorithms: a genome-wide benchmark
Source: Nucleic Acids Res. 2015 Jan 23;43(3):1859–68. doi: 10.1093/nar/gkv010 (PMC4330374; doi:10.1093/nar/gkv010)
Supplement: SUPPLEMENTARY DATA [file supp_gkv010_nar-02823-z-2014-File007.pdf]

Supplementary Material

Sequence Files:
Text files containing the riboSNitch sequences used in this benchmark are provided by category. These are named “probedRibos.txt”, “validatedRibos.txt”, “symmetricRibos.txt”, “asymmetricRibos.txt” and “allRibos.txt.” The 1058 non-riboSNitch sequences are provided in the file “nonribos.txt”

Table S1. AUC values for algorithms’ ROC performance on the entire riboSNitch dataset and two subsets—the best 25% and best 5% as ranked by riboSNitch average fdr-adjusted p-value. 95% confidence intervals shown below each AUC value.

Algorithm	*All*	*Best 25%*	*Best 5%*
CentroidFold	0.532<br>(0.507-0.556)	0.565<br>(0.517-0.614)	0.552<br>(0.440-0.664)
CONTRAfold	0.535<br>(0.510-0.559)	0.566<br>(0.517-0.615)	0.547<br>(0.435-0.659)
MC-Fold	0.493<br>(0.464-0.522)	0.483<br>(0.439-0.528)	0.487<br>(0.381-0.563)
remuRNA	0.537<br>(0.513-0.562)	0.560<br>(0.532-0.613)	0.579<br>(0.480-0.637)
RNAfold	0.534<br>(0.509-0.559)	0.557<br>(0.504-0.585)	0.537<br>(0.424-0.576)
RNAmute	0.511<br>(0.487-0.535)	0.508<br>(0.466-0.538)	0.455<br>(0.386-0.513)
RNAmutants	0.509<br>(0.485-0.533)	0.502<br>(0.464-0.539)	0.417<br>(0.397-0.540)
RNAsnp	0.533<br>(0.508-0.558)	0.564<br>(0.512-0.593)	0.561<br>(0.453-0.617)
RNAstructure	0.536<br>(0.511-0.560)	0.543<br>(0.502-0.582)	0.526<br>(0.440-0.588)
SNPfold	0.528<br>(0.504-0.553)	0.553<br>(0.494-0.574)	0.525<br>(0.416-0.570)
UNAFold	0.526<br>(0.502-0.551)	0.543<br>(0.493-0.592)	0.530<br>(0.418-0.643)

Table S2. The sensitivity and specificity values and corresponding threshold of the most top left point in each ROC curve.

Category		Centroid Fold	contra Fold	MC-Fold	remu RNA	RNAfold	RNAmute	RNAmut ants	RNAsnp	RNAstru cture	SNPfold	UNAFold
**Probed**	Threshold	0.44	0.79	6.00	2.82	3.97	19.00	20.00	0.39	1.77	0.92	3.08
	Sensitivity	0.82	0.64	0.67	0.64	0.73	0.55	0.64	0.83	0.73	0.73	0.73
	Specificity	0.45	0.64	0.38	0.82	0.73	0.64	0.64	0.73	0.55	0.73	0.45
**Validated**	Threshold	0.89	0.62	44.00	1.65	4.48	6.00	11.00	0.30	3.14	0.93	2.22
	Sensitivity	0.56	0.56	0.34	0.56	0.59	0.62	0.46	0.48	0.51	0.60	0.59
	Specificity	0.60	0.48	0.80	0.56	0.62	0.51	0.52	0.75	0.63	0.60	0.52
**Symmetric**	Threshold	0.75	0.60	7.00	2.38	5.22	5.00	7.00	0.47	2.76	0.93	2.68
	Sensitivity	0.55	0.49	0.52	0.45	0.50	0.54	0.48	0.56	0.48	0.54	0.50
	Specificity	0.58	0.42	0.49	0.64	0.65	0.53	0.53	0.59	0.62	0.61	0.60
**Asymmetric**	Threshold	0.71	0.50	17.00	1.68	3.16	5.00	5.00	0.49	2.52	0.95	1.98
	Sensitivity	0.52	0.54	0.45	0.50	0.58	0.54	0.51	0.53	0.48	0.54	0.52
	Specificity	0.52	0.44	0.56	0.56	0.47	0.51	0.51	0.53	0.57	0.49	0.53
**All**	Threshold	0.65	0.50	17.00	1.65	3.73	5.00	5.00	0.47	2.52	0.94	2.09
	Sensitivity	0.55	0.52	0.45	0.51	0.53	0.54	0.50	0.52	0.49	0.55	0.51
	Specificity	0.49	0.44	0.56	0.55	0.52	0.50	0.51	0.55	0.58	0.50	0.54
**25% tails**	Threshold	0.106	0.068	31.000	4.711	0.224	31.000	31.000	0.084	10.479	0.674	8.748
	Sensitivity	0.631	0.573	0.358	0.539	0.605	0.257	0.252	0.527	0.438	0.549	0.487
	Specificity	0.548	0.517	0.635	0.370	0.556	0.765	0.754	0.599	0.668	0.588	0.582
**5% tails**	Threshold	0.017	2.219	42.000	7.192	17.319	42.000	42.000	0.999	9.637	0.999	0.005
	Sensitivity	0.636	0.608	0.124	0.500	0.686	0.065	0.062	0.808	0.615	0.811	0.667
	Specificity	0.620	0.604	0.878	0.326	0.679	0.940	0.924	0.559	0.615	0.647	0.500

Table S3. AUC values with 95% confidence intervals for algorithms tested with a Pearson correlation coefficient metric. Previous values from the RNApdist metric shown for comparison.

Software	*Metric*	*Probed*	*Validated*	*Symm*	*Asymm*	*All*
CentroidFold	RNApdist	0.579 (0.324-0.833)	0.561 (0.460-0.662)	0.569 (0.516-0.622)	0.529 (0.502-0.557)	0.532 (0.507-0.556)
	Pearson	0.628 (0.384-0.872)	0.559 (0.458-0.66)	0.563 (0.51-0.616)	0.527 (0.500-0.555)	0.530 (0.505-0.554)
CONTRAfold	RNApdist	0.463 (0.195-0.73)	0.562 (0.461-0.664)	0.567 (0.514-0.62)	0.528 (0.500-0.556)	0.535 (0.510-0.559)
	Pearson	0.587 (0.334-0.840)	0.573 (0.472-0.674)	0.568 (0.515-0.621)	0.532 (0.504-0.559)	0.538 (0.513-0.562)
RNAstructure	RNApdist	0.612 (0.358-0.865)	0.578 (0.478-0.678)	0.567 (0.513-0.62)	0.527 (0.499-0.554)	0.536 (0.511-0.560)
	Pearson	0.603 (0.354-0.852)	0.561 (0.461-0.663)	0.546 (0.492-0.599)	0.522 (0.494-0.549)	0.529 (0.505-0.554)
UNAFold	RNApdist	0.471 (0.210-0.732)	0.537 (0.435-0.639)	0.548 (0.494-0.601)	0.524 (0.496-0.551)	0.526 (0.502-0.551)
	Pearson	0.455 (0.186-0.723)	0.518 (0.416-0.620)	0.526 (0.473-0.580)	0.517 (0.489-0.545)	0.520 (0.495-0.544)

Table S4. Parameters used for each algorithm.

Algorithm	*Unix Command Parameters*
CentroidFold	$ centroid_fold --engine McCaskill -o output.txt --posteriors 0 seq.fa
CONTRAfold<br>(for MFE)	$ ./contrafold predict --posteriors 0 output.txt seq.fa<br>$ ./contrafold predict --parens output.txt seq.fa
MC-Fold	$ export QUERY_STRING="pass=lucy&sequence="$seq"&top=1"<br>$ ./mcfold.static.exe
remuRNA	$ ./remuRNA seq.fa
RNAfold<br>(for MFE)	$ cat seq.fa | RNAfold –p<br>$ cat seq.fa | RNAfold
RNAmutants	$ ./RNAmutants -l ~/programs/RNAmutants-master/lib -f seq.fa --mutation 0 --sample-number 0
RNAmute	$ java -jar RNAmute.jar seq.txt
RNAsnp	$ ./RNAsnp -f seq.fa -s snp.txt -w 100 -l 10 -c 0.0
RNAstructure<br>(for MFE)	$ partition seq.fa out.pfs<br>$ Fold -mfe seq.fa out.ct
SNPfold	$ SNPfold_commandline.py "$seq" "$ref"51"$alt"
UNAFold<br>(forMFE)	$ UNAFold.pl --model=PF seq.fa<br>$ hybrid-ss-min --suffix DAT seq.fa

Figure S1. ROC performance of selected algorithms (A-D) on different input sequence lengths from the "probed" category of riboSNitches. Sequence lengths ranged from 21 to 201 in 6 base increments. The graphs indicate large variance with sequence length, although for some algorithms, performance remains robust within certain sub-ranges.

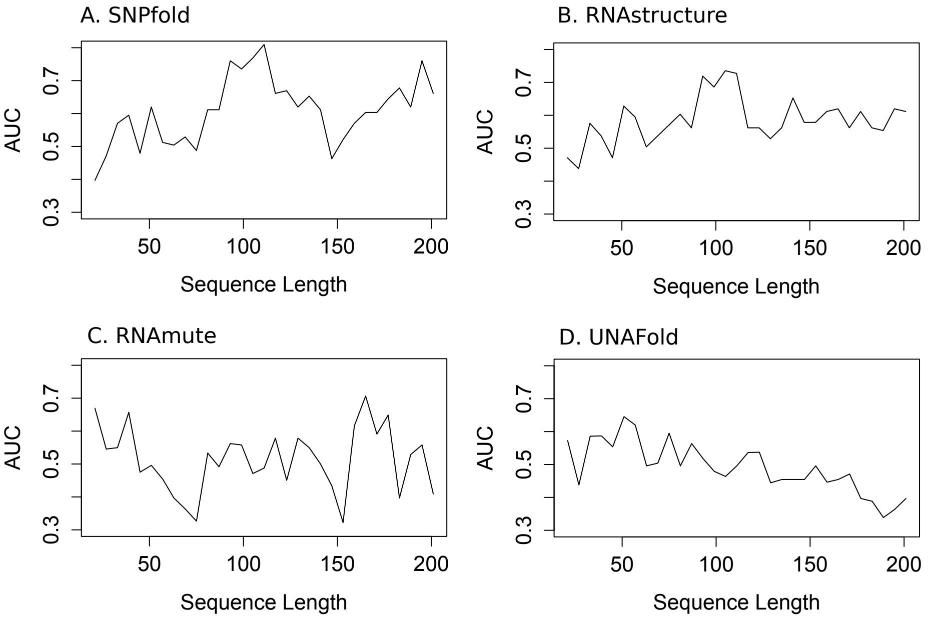

Figure S2. ROC performance across riboSNitch categories on a range of sequence lengths for SNPfold, RNAstructure and a random function (A-C). The random function sampled scores from the combined set of all SNPfold riboSNitch and non-riboSNitch scores. The trend of decreasing AUC values from the "probed" category to the "all" category remains evident with SNPfold and RNAstructure and is absent in the more symmetrical random function.

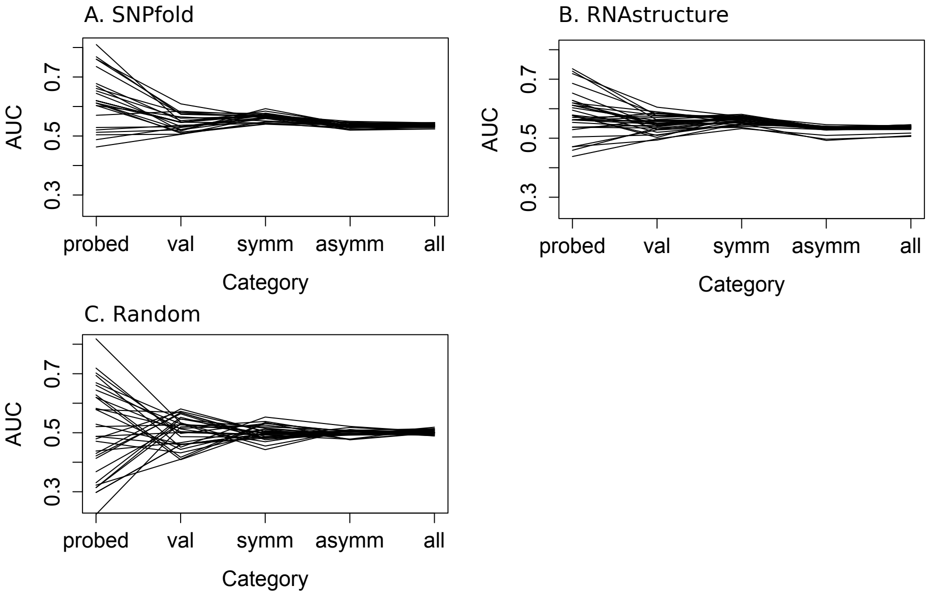

Figure S3. Algorithms' distance score distributions for all riboSNitches and matched non-riboSNitches. The 5% tails of the distributions are shaded grey, though are too small to see in many cases. remuRNA returns the relative entropy between variant RNAs, which can be negative. SNPfold returns Pearson correlation coefficients instead of true distance scores and RNAsnp returns p-values. The structure distance functions RNAdistance and RNApdist were used with the rest of the algorithms, generating distance scores greater than or equal to zero. **A)** Score distributions for specialized algorithms. **B)** Score distributions for general algorithms.

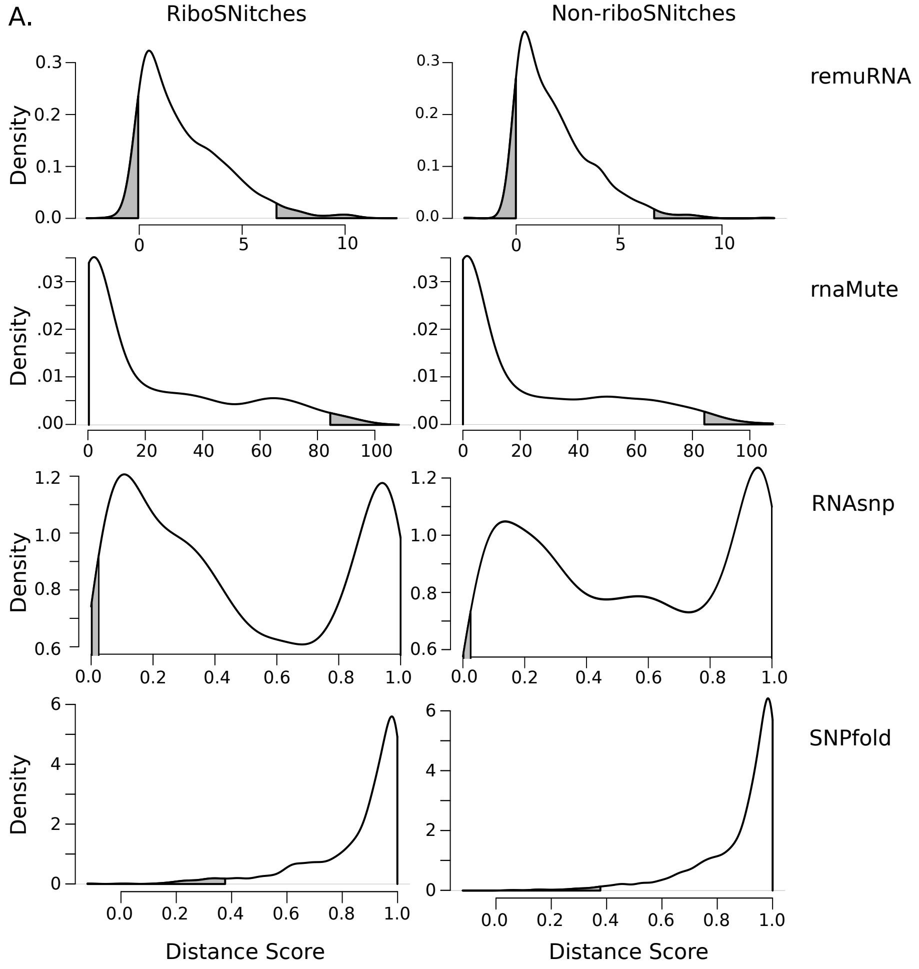

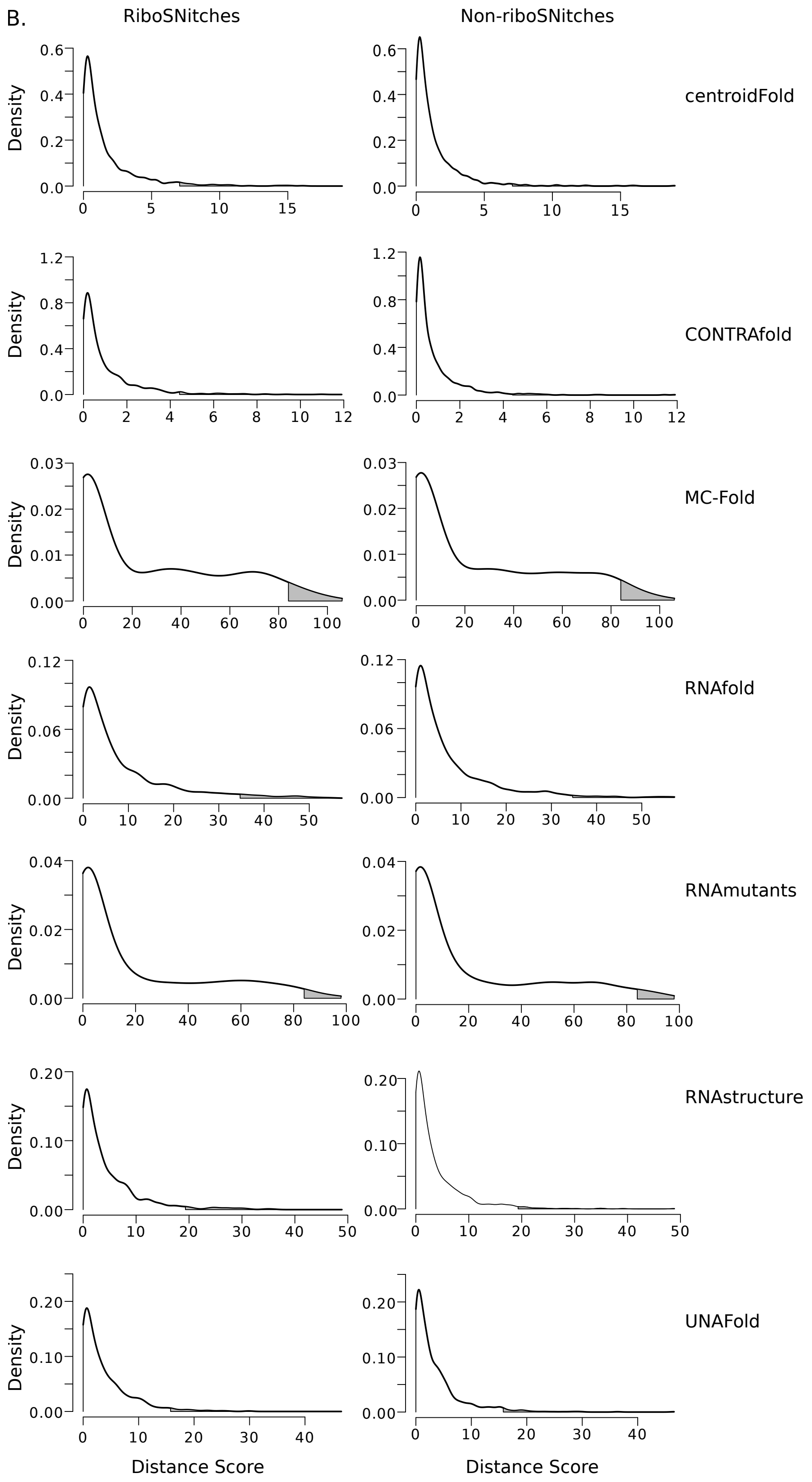
B.
RiboSNitches
Non-riboSNitches
centroidFold
CONTRAfold
MC-Fold
RNAfold
RNAmutants
RNAstructure
UNAFold
Density
Distance Score
